# How High Can You Get: A Case Report of an Unusual Cause of Ascending Paralysis

**DOI:** 10.7759/cureus.21236

**Published:** 2022-01-14

**Authors:** Ayrton I Bangolo, Sarah Pender, Chandini Sajja, Daniel Matassa, Benjamin Perrella

**Affiliations:** 1 Internal Medicine, Palisades Medical Center, North Bergen, USA; 2 Internal Medicine, University Hospital - Rutgers New Jersey Medical School, Newark, USA

**Keywords:** end stage renal disease (esrd), case report, electrolytes, dialysis, paralysis

## Abstract

Secondary hyperkalemic paralysis is a life-threatening manifestation of hyperkalemia seen with a potassium level of 7 or above 7 milliequivalents per liter (Meq/L) in an acute or chronic state. Standard hyperkalemic treatment should be initiated upon diagnosis with emergency dialysis in refractory cases. Here we present the case of a patient with end-stage renal disease (ESRD) compliant with dialysis three times a week. The patient presented with generalized ascending flaccid paralysis and was found to have serum potassium of 9.6 Meq/L. Spontaneous resolution of the paralysis was observed shortly after the completion of one hemodialysis session. The goal of this case report is to raise awareness of a life-threatening complication of electrolyte imbalances in ESRD even in patients that are compliant with dialysis.

## Introduction

Hyperkalemia-induced ascending paralysis secondary to familial hyperkalemic periodic paralysis (HYPP) is well described; however, secondary hyperkalemic paralysis in the absence of an underlying genetic disorder has only been described in a handful of case reports [1]. HYPP is an autosomal dominant disease with episodes of myopathic weakness typically presenting before the age of 20, lasting 15 minutes to an hour, and triggered by increased potassium intake or rest after heavy exercise [1,2]. Secondary hyperkalemia can cause muscle weakness that may progress to paralysis, starting with the lower extremities and progressing through the trunk and upper extremities mimicking Guillain-Barre syndrome [3]. Sphincter tone and cranial nerves are spared with minimal sensory deficits [3]. Hyperkalemia is a common problem encountered in chronic kidney disease when the patient becomes oliguric or has additional problems such as a high potassium diet, increased tissue breakdown, or hypoaldosteronism [4]. Here we present the case of a patient with end-stage renal disease (ESRD) on dialysis who developed life-threatening secondary hyperkalemic paralysis secondary to high potassium intake.

## Case presentation

This is a 61-year-old male with a past medical history significant for hypertension and ESRD on dialysis thrice weekly for the past seven years who presented for evaluation of ascending lower and upper extremities weakness. The weakness was sudden in onset, progressive, and without remittance. It began shortly after he reported a mechanical fall. The patient’s last dialysis session was two days prior. Most notably, he endorsed an increased consumption of bananas over the past few days because he was experiencing lower extremity muscle cramps.

**Figure 1:**
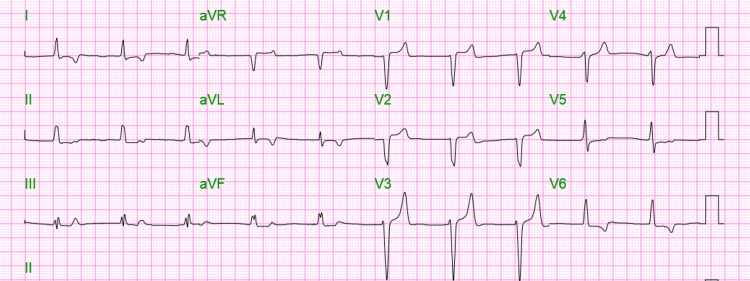
Junctional rhythm with atypical left bundle branch block and prominent T waves.

**Figure 2:**
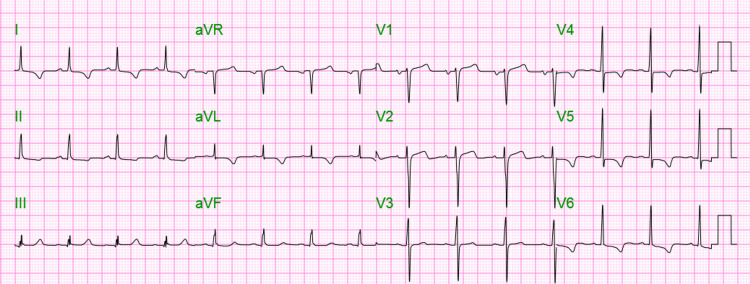
Sinus rhythm with inverted T waves in lateral leads.

On physical examination, there were diminished breath sounds throughout the entire lung fields, the abdomen was soft but moderately tender in all four quadrants, and there was complete paralysis of all four extremities (motor strength 0/5); areflexia but without cranial nerve or sensory deficits. His initial chemistry panel revealed a blood urea nitrogen of 41 milligrams per deciliter (mg/dl) (7-25), creatinine of 5.89 mg/dl (0.70-1.30), and potassium of 9.6 millimoles per liter (mmol/L) (3.5-5.1). The initial EKG revealed a junctional rhythm with an atypical left bundle branch block and prominent T waves (Figure 1). A head CT did not reveal any acute pathologies. The patient received calcium gluconate and regular insulin intravenously. Emergent dialysis was performed. Following dialysis, the paralysis had resolved and the repeat chemistry revealed potassium of 7.4 mmol/L. The repeat EKG revealed a sinus rhythm with inverted T waves in lateral leads and resolution of the bundle branch block (Figure 2). The patient received a second dialysis session the following day, after which the potassium decreased to 5.4 mmol/L. He was discharged home with instructions to continue his scheduled dialysis sessions and follow a lower potassium dietary intake.

## Discussion

Severe hyperkalemia, defined as serum potassium above 6 millimoles per liter (mmol/L) is seen in approximately 1% of hospitalized patients, with a significant risk of mortality. Excessive intake of potassium is a rare cause of symptomatic hyperkalemia given the body’s adaptive capacity to increase renal secretion; however, dietary intake can have a major effect in susceptible patients such as those with ESRD [1]. The most serious complications of hyperkalemia are muscle weakness, paralysis, cardiac conduction abnormalities, and life-threatening cardiac arrhythmias [3]. The skeletal muscle and cardiac manifestations of hyperkalemia typically have one or more EKG changes associated with hyperkalemia [3].

Secondary hyperkalemic paralysis is flaccid and ascending, mimicking Guillain-Barre. However, secondary hyperkalemic paralysis does not typically cause rectal tone dysfunction or respiratory muscle paralysis [3], which was preserved in our patient.

A diagnosis of secondary hyperkalemic paralysis can be challenging given the wide variety of causes of flaccid paralysis, and early EKG changes and elevated serum potassium can assist in making the diagnosis, especially in a patient with chronic kidney disease. Although early EKG changes are often seen with profound serum potassium elevation, a case series revealed two cases of severe hyperkalemia with serum potassium above 9 mmol/L without expected EKG manifestations of hyperkalemia [5]. Thus, it is especially important to keep in mind that a normal EKG does not exclude severe hyperkalemia; confirmation of hyperkalemia with a chemistry panel should not be delayed.

All patients should receive standard hyperkalemic treatment consisting of calcium gluconate with EKG changes or severe hyperkalemia to stabilize cardiac myocytes. Beta-agonists or intravenous insulin should be given to promote the intracellular shift of potassium in conjunction with medications that promote the increase of potassium excretion from the body [6]. Hemodialysis is the most effective and reliable method to reduce serum potassium concentration; peritoneal dialysis is considered less effective [1,6]. Initiation of dialysis can often take several hours; therefore, even if dialysis is contemplated, standard hyperkalemic treatment should be initiated as a bridge to dialysis [7]. Our patient was administered standard therapy while awaiting hemodialysis.

## Conclusions

Secondary hyperkalemic paralysis is a rare manifestation of hyperkalemia. This case report reveals a diagnostic differential that should be considered as a potential etiology of ascending flaccid paralysis, especially in patients with impaired renal function and an elevated potassium level. We also emphasize the importance of educating patients with ESRD about maintaining a low potassium diet and compliance with dialysis to prevent life-threatening electrolyte imbalances.
